# Case Report: A Case of Intestinal Behçet's Disease Exhibiting Enhanced Expression of IL-6 and Forkhead Box P3 mRNA After Treatment With Infliximab

**DOI:** 10.3389/fmed.2021.679237

**Published:** 2021-05-14

**Authors:** Keisuke Yoshikawa, Tomohiro Watanabe, Ikue Sekai, Ryutaro Takada, Akane Hara, Masayuki Kurimoto, Yasuhiro Masuta, Yasuo Otsuka, Tomoe Yoshikawa, Sho Masaki, Ken Kamata, Kosuke Minaga, Yoriaki Komeda, Takaaki Chikugo, Masatoshi Kudo

**Affiliations:** ^1^Department of Gastroenterology and Hepatology, Kindai University Faculty of Medicine, Osaka, Japan; ^2^Department of Diagnostic Pathology, Kindai University Hospital, Osaka, Japan

**Keywords:** intestinal Behçet's disease, IL-6, Foxp3, infliximab, regulatory T cells

## Abstract

Behçet's disease (BD) is a rare inflammatory condition characterized by oral and genital ulcers, skin lesions, as well as ophthalmological, neurological, and gastrointestinal manifestations. BD involving the gastrointestinal tract is known as intestinal BD. The mucosa of the gastrointestinal tract of patients with intestinal BD exhibits enhanced levels of proinflammatory cytokines, such as IL-1β, IL-6, and TNF-α. These proinflammatory cytokines play pathogenic roles in the development of BD, as evidenced by the fact that biologics targeting these cytokines effectively induce BD remission. It should be noted, however, that the molecular mechanisms by which the blockade of these cytokines suppresses chronic inflammatory responses in BD are poorly understood. Herein, we report a case of intestinal BD resistant to prednisolone that was successfully treated with infliximab (IFX). The induction of remission by IFX was accompanied by a marked elevation of IL-6 and forkhead box P3 (FOXP3) at mRNA level. This case suggests that induction of remission by IFX is mediated not only by the suppression of TNF-α-mediated signaling pathways, but also by the promotion of IL-6 expression and accumulation of regulatory T cells expressing FOXP3.

## Introduction

Behçet's disease (BD) is a rare inflammatory condition characterized by oral and genital ulcers, skin lesions, and patients with BD exhibit ophthalmological, neurological, and gastrointestinal manifestations (1). Approximately 5–15% of patients with BD are diagnosed with intestinal BD, and they usually present with abdominal pain and diarrhea (1, 2). A typical endoscopic finding in patients with intestinal BD is a giant, oval-shaped, deep punched-out ulcer in the ileocecal area (1). The active phase of BD is characterized by high T helper 17 (Th17)/regulatory T cell (Treg) ratio (3, 4). In addition to the involvement of Th17 and Treg cells, increased levels of the proinflammatory cytokines, such as IL-1β, IL-6, and TNF-α, underlie the immunopathogenesis of BD, including that of intestinal BD (1, 5). This idea is supported by the clinical success of biologics targeting proinflammatory cytokines in patients with BD (1, 5, 6). For example, administration of infliximab (IFX) and anakinra, which neutralize TNF-α and IL-1β, respectively, led to remission even in patients with BD refractory to prednisolone (PSL) (1, 5, 6). It should be noted, however, that the molecular mechanisms underlying the suppression of chronic inflammatory responses in intestinal BD are poorly understood. It is possible that the blockade of proinflammatory cytokines is not limited to the suppression of signaling pathways specific to each cytokine. Indeed, the neutralization of TNF-α by IFX or adalimumab does not always suppress TNF-α-mediated signaling pathways, but also promote the accumulation of Tregs expressing forkhead box P3 (FOXP3) (7, 8). Herein, we report a case of intestinal BD refractory to PSL that was successfully treated with IFX. The induction of remission by IFX in this case was accompanied by a marked elevation of IL-6 and FOXP3.

## Case Description

A 69-year old man was admitted for further examination of bloody diarrhea, appetite loss, and high-grade fever (>38°C), that persisted ~1 month. He was diagnosed with uveitis and treated at the Department of Ophthalmology. On admission, he developed periodic fever accompanied by bloody diarrhea. Physical examination revealed oral aphthous ulcers without genital ulcers or erythema nodosum. Leukocytosis (white blood cell count: 10,200/μL) and anemia (hemoglobin: 9.1 g/dL) were detected in complete blood cell counts. Although, the liver and kidney function parameters were within normal limits, severe hypoalbuminemia (albumin: 1.7 g/dL, normal range 4.1–5.1) was seen. Serum concentrations of C-reactive protein (8.717 mg/dL, normal range <0.14) and IgG (2,492 mg/dL, normal range 861–1,747) were elevated. The patient's human leukocyte antigen was B54, and neither anti-neutrophil cytoplasmic antibody nor anti-nuclear antibody was positive. No pathogenic bacteria were detected in blood or stool cultures.

## Diagnostic Assessment

**Figure 1:**
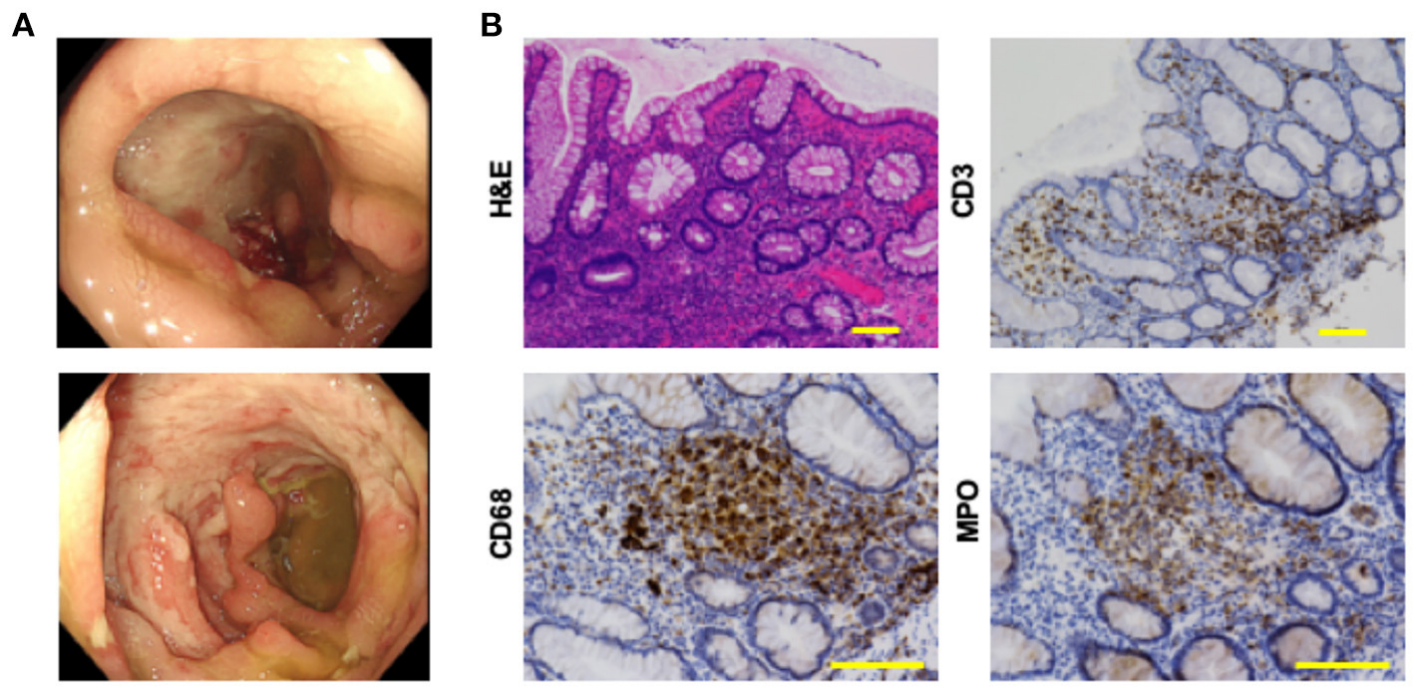
Colonoscopic and pathological findings in a patient with intestinal Behçet's disease before the treatment. **(A)** Colonoscopy revealed punched-out, deep and semi-circumferential ulcers in the cecum (top), and sigmoid colon (bottom), respectively. **(B)** Colonic biopsy samples were subjected to hematoxylin and eosin (H&E) staining and immunohistochemical analysis to visualize CD3^+^ T cells, CD68^+^ macrophages, and myeloperoxidase (MPO)^+^ granulocytes. Accumulation of immune cells and destruction of crypt architecture were observed in tissues stained by H&E. Colonic mucosa of this patient with intestinal Behçet's disease was characterized by the infiltration of CD3^+^ T cells, CD68^+^ macrophages, and MPO^+^ granulocytes. Scale bar, 100 μm.

Whole body computed tomography was performed to identify the causes of bloody diarrhea and periodic fever. Diffuse wall thickening of the ascending and transverse colon was detected on abdominal computed tomography. Deep, punched-out, oval-shaped, and semi-circumferential ulcers were observed in the cecum and sigmoid colon, respectively, during colonoscopy (Figure 1A). Pathological examination revealed destruction of the crypt architecture and massive accumulation of lymphocytes and granulocytes in the colonic lamina propria (Figure 1B). Granuloma formation or crypt abscesses were not observed. Polymerase chain reaction (PCR) tests for the detection of tuberculosis or cytomegalovirus using colonic biopsy samples were negative. These endoscopic and pathological findings were not consistent with Crohn's disease or ulcerative colitis. Based on the presence of uveitis, oral ulcers, and intestinal symptoms, the patient was diagnosed with intestinal BD according to the international diagnostic criteria for BD (2).

Colonic biopsy samples were subjected to immunohistochemical analysis, as previously described (9, 10). Briefly, deparaffinized sections were incubated with rabbit anti-human CD3 monoclonal antibody (mAb, clone 2GV6, Roche Diagnostics), mouse anti-human CD20 mAb (clone L26, Roche Diagnostics), rabbit anti-human polyclonal myeloperoxidase (MPO)^+^ Ab (#518-114428, Roche Diagnostics) and mouse anti-human CD68 mAb (clone KP-1, Agilent Technology) followed by visualization of protein expression by Dako EnVision^+^ System (Agilent Technology). Primary Ab dilution was done according to the recommendation of the manufacturers. Immunohistochemical photographs were captured by microscopy (Biozero, BZ-8100, Keyence). As shown in Figure 1B, the colonic mucosa was characterized by massive accumulation of CD3^+^ T cells, CD68^+^ macrophages, and MPO^+^ granulocytes, but not of CD20^+^ B cells (data not shown).

**Figure 2:**
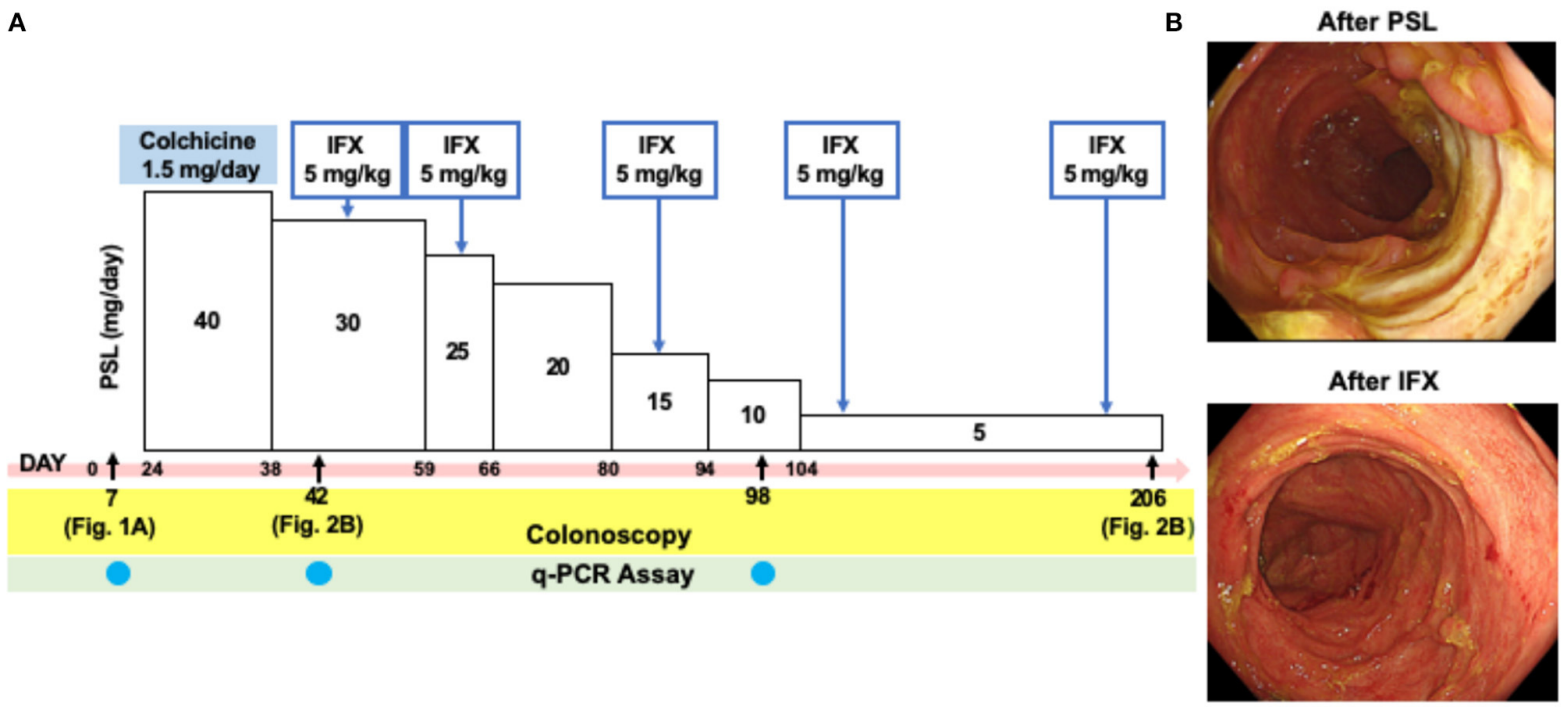
Colonoscopic findings in a patient with intestinal Behçet's disease after the treatment with prednisolone and infliximab. **(A)** Clinical course of a patient with intestinal Behçet's disease. Day 0 was defined as on day of admission. **(B)** Colonoscopy performed after the treatment with prednisolone (PSL, Day 42) revealed semi-circumferential ulcers in the sigmoid colon. Disappearance of multiple ulcers was seen in the sigmoid colon after five times injections of infliximab (IFX, Day 206).

The initial treatment with oral colchicine (1.5 mg/day) based on the diagnosis of intestinal BD was unsuccessful. The patient was then treated with oral PSL (40 mg/day) with a tapering schedule as shown in the clinical course (Figure 2A), which again failed to improve his symptoms. As shown in Figure 2B (top panel), active ulcers were still present in the cecum and sigmoid colon. IFX (5 mg/kg) was then administered to induce remission. Five injections of IFX, which was tolerable without adverse events, led to complete remission in this case, as indicated by the complete disappearance of colonic ulcers through colonoscopy, and by improvement of the patient's symptoms (Figure 2B, bottom panel). Thus, this case of intestinal BD was successfully treated with IFX.

## Quantitative PCR Analyses

Total mRNA was isolated from colonic biopsy samples during colonoscopy and subjected to quantitative PCR (qPCR) analyses as previously described (10). mRNA was isolated from colonic biopsy samples by using Monarch Total RNA Miniprep kit (New England Biolabs) and then was reverse transcribed into cDNA by High-Capacity RNA to cDNA kit (Applied Biosystems). The mRNA level for each target gene was determined by SYBR Green-based qPCR using a Step One Plus system (Applied Biosystems) and Quantitect primer assays (Qiagen) followed by the normalization using β-actin as a reference gene. Each PCR primer for Quantitect primer assays was purchased from Qiagen as follows; β-actin (#QT00095431), TNF-α (#QT00029162), IFN-β (#QT000203763), IFN-γ (#QT00000525), IL-6 (#QT00083720), IL-9 (#QT00000630), IL-12 (#QT00000364), IL-13 (#QT00000511), IL-33 (#QT00041559), C-C chemokine ligand 17 (CCL17, #QT00096866), CCL26 (#QT00023135), FOXP3 (#QT00048286), C-X-C motif chemokine ligand 9 (CXCL9, #QT00013461), and CXCL10 (#QT01003065). In some experiments, custom made primers were obtained from Invitrogen. Primer sequences were as follows; IL-1β (sense; GGACAAGCTGAGGAAGATGC, antisense; TCGTTATCCCATGTGTCGAA), IL-5 (sense; TGAGGATGCTTCTGCATTTG, antisense; GCAGTGCCAAGGTCTCTTTC), IL-17 (sense; CATGAACTCTGTCCCCATCC, antisense; CCCACGGACACCAGTATCTT), IL-23 (sense; TCTCCTTCTCCGCTTCAAAA, antisense; TTAGGGACTCAGGGTTGCTG). Quality of RNA and cDNA was checked by NanoDrop (Thermo Fisher Scientific). Annealing temperature was optimized and the specificity of the reaction was confirmed by the presence of a sharp single peak in melting curve analyses. mRNA expression levels of target genes were normalized by that of β-actin mRNA by using Ct method. Thus, qPCR analyses were performed in accordance with the MIQE guidelines.

**Figure 3:**
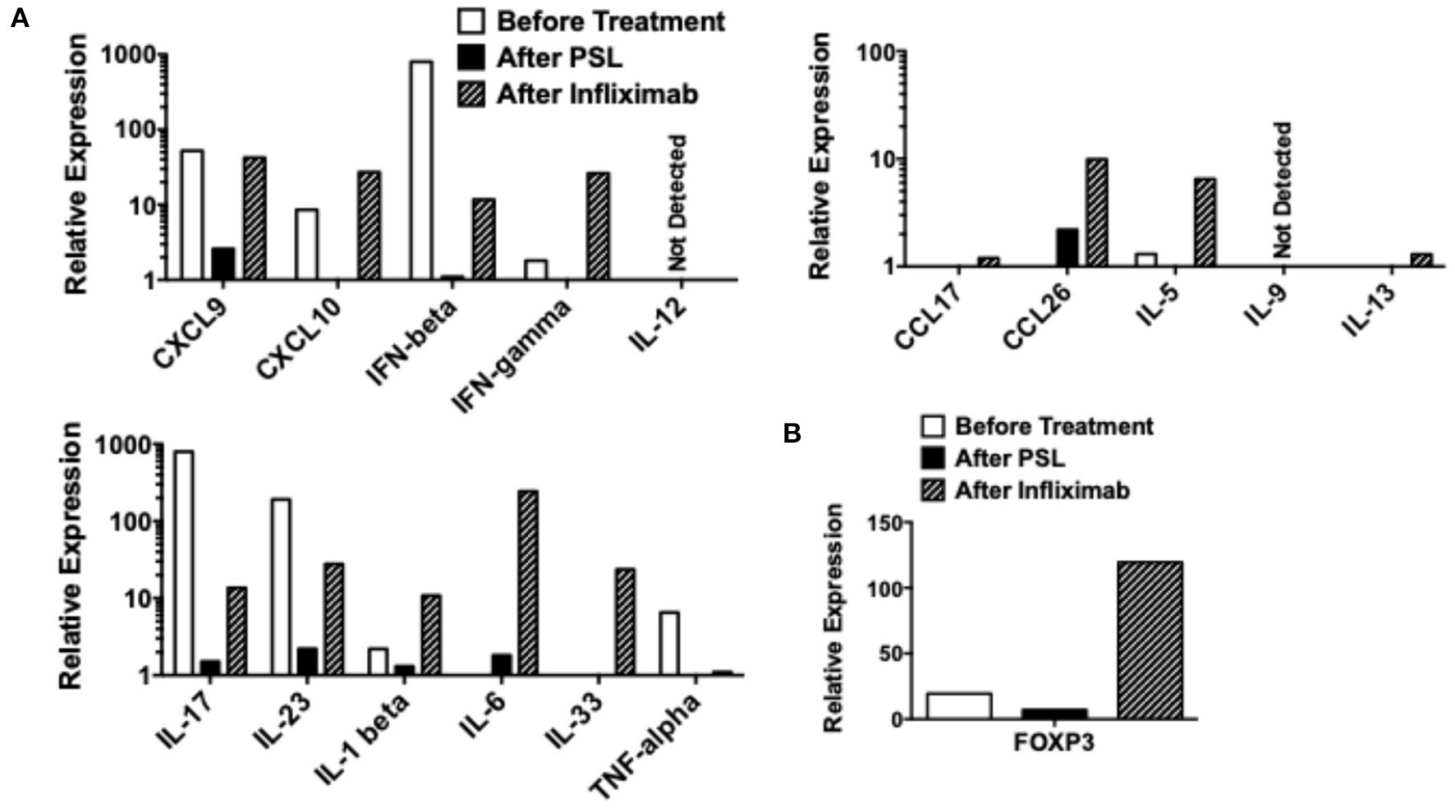
Quantitative polymerase chain reaction analyses in colonic biopsy samples. Colonic biopsy samples were subjected to quantitative polymerase chain reaction analyses to determine expression of cytokines and chemokines. Colonic biopsy samples were obtained before the treatment (Day 7 in Figure 2A), after the treatment with prednisolone (PSL, Day 42 in Figure 2A), and after the treatment with infliximab (IFX, Day 98 in Figure 2A). mRNA expression levels of cytokines, chemokines **(A)**, and forkhead box P3 (FOXP3) **(B)** were normalized by that of β-actin mRNA.

The levels of mRNAs encoding proinflammatory cytokines and chemokines were compared in samples isolated before treatment (Day 7), after treatment with PSL (Day 42), and after three injections of IFX (Day 98). qPCR analyses revealed that the colonic mucosa before treatment was characterized by elevated mRNA expression levels of IL-17, IL-23, CXCL9, CXCL10, and IFN-β (Figure 3A). Although, treatment with PSL led to a marked decrease in all proinflammatory cytokines and chemokines at mRNA level, remission was not achieved. Notably, successful induction of remission by IFX was accompanied by elevated mRNA expression levels of CXCL9, CXCL10, IFN-β, IFN-γ, IL-1β, IL-6, and IL-33 as compared with those after PSL treatment. IL-6 mRNA expression was particularly strongly induced by IFX, suggesting that IL-6 may be involved in the improvement of chronic inflammation in this case. Expression of colitogenic pro-inflammatory cytokines at mRNA level, such as IL-17, IL-23, and TNF-α were suppressed by both PSL and IFX treatments, compared to their levels before treatment. We also examined the mRNA expression of FOXP3 and found that treatment with IFX markedly increased its levels in the colonic mucosa (Figure 3B). Thus, increased expression of IL-6 and FOXP3 and decreased expression of TNF-α at mRNA level might be key factors in the resolution of severe inflammation in this case of intestinal BD.

## Discussion

Here, we report a case of intestinal BD that was successfully treated with IFX. This case was diagnosed as intestinal BD according to the international diagnostic criteria for BD (2). Our qPCR analyses of colonic biopsy samples revealed that the colonic mucosa of this patient was characterized by increased expression levels of IL-17, IL-23, TNF-α, IFN-β, CXCL9, and CXCL10 before treatment. Treatment with PSL decreased cytokine and chemokine expression levels in our qPCR analyses, which, however, did not result in the induction of remission. Interestingly, treatment switch from PSL to IFX led to complete remission of intestinal BD. The induction of complete remission by IFX was likely related to the marked increase in the expression levels of IL-6 and FOXP3 mRNA caused by this biologic drug, which was concomitant with the suppressed expression of IL-17, IL-23, and TNF-α mRNA.

Given that activation of IL-6-mediated signaling pathways underlies the immunopathogenesis of various autoimmune diseases, the increase in IL-6 expression by IFX was paradoxical (11). Indeed, serum concentrations of IL-6 are higher in patients with BD than in healthy controls (5). In this regard, Rakoff-Nahoum et al. (12) showed that regeneration of the intestinal epithelium depends upon the activation of IL-6-mediated signaling pathways in an experimental model of colitis. In this case, regeneration of the intestinal epithelium was promoted soon after treatment with IFX. Therefore, we hypothesize that the induction of IL-6 expression contributed to the remission of BD by promoting epithelial regeneration. However, we need to have a thorough understanding regarding the exact role played by IL-6 in BD, because tocilizumab, an anti-IL-6 receptor Ab, is effective in a significant fraction of patients with refractory uveitis associated with BD (13). Further, studies addressing molecular mechanisms accounting for the link between IL-6 and remission are therefore required to determine the beneficial roles of IL-6 in patients with BD.

Another important feature observed in our qPCR analyses was the increased expression of FOXP3, a critical transcription factor in Tregs (14). Activation of FOXP3^+^ Tregs suppresses chronic inflammatory reactions (14). IFX treatment was accompanied by elevated FOXP3 expression in the colonic mucosa. Given the fact that Tregs expressing FOXP3 suppress colonic inflammatory responses, it is likely that induction of remission was partially achieved by the accumulation of Tregs in this case of intestinal BD (14). In agreement with this notion, neutralization of TNF-α by IFX or adalimumab led to increased expression of FOXP3 and accumulation of Tregs (7, 8). In this regards, augmented IL-33 responses induced by IFX treatment might be also involved in the FOXP3 expression since this cytokine has the ability to promote differentiation of Tregs in the gastrointestinal tract (15). All-trans retinoic acid, a metabolite of Vitamin A, induces differentiation of Tregs in the gastrointestinal tract (16). Therefore, Vitamin A in the diet might promote Treg differentiation. However, the relationship between Vitamin A uptake and BD pathogenesis has been poorly defined.

In contrast to the results of the IFX treatment, the treatment with PSL markedly reduced expression levels of all cytokines and chemokines in our qPCR analyses. Furthermore, PSL treatment decreased FOXP3 mRNA expression, indicating that PSL suppressed a broad range of immune responses. Consistent with these data, Ogawa et al. (17) provided evidence that PSL inhibits nuclear translocation of nuclear factor κB and interferon regulatory factors, thereby suppressing the production of cytokines and chemokines. In addition to the suppression of typical colitogenic cytokines such as IL-17, IL-23, and TNF-α (18), treatment with PSL notably diminished type I IFN responses (IFN-β, CXCL9, and CXCL10). Given that type I IFN cytokines and chemokines constitute a part of mucosal host defense against gut microorganisms, the failure of PSL to induce remission might be partially explained by the impaired mucosal host defenses due to the impaired activation of type I IFN responses (19). Thus, mechanisms of action differ between PSL and IFX, in that the latter promotes the expression of IL-6 and FOXP3, whereas, the former suppresses almost all immune responses. Such differences likely explain the observation that IFX was able to successfully induce remission, whereas, PSL lacked this therapeutic ability.

Treatment with IFX led to enhanced expression of IL-6 and FOXP3 at mRNA level, but not protein level. Thus, we have not confirmed expression of IL-6 or FOXP3 at protein level. Given that IL-6 and FOXP3 are subjected to post-transcriptional and post-translational processes, it is possible that increased expression of IL-6 and FOXP3 at mRNA level does not lead to functional expression at protein level. We have to be cautious regarding the interpretation of increased expression of IL-6 and FOXP3 at mRNA level. Therefore, further studies addressing expression of IL-6 and FOXP3 at protein level are absolutely required to verify our idea that enhanced expression of IL-6 and FOXP3 is associated with the induction of remission of intestinal BD by IFX. In addition, we cannot completely exclude a possibility that cytokines other than IL-6, e.g., TGF-β1 and IL-10, might play beneficial roles in the resolution of chronic intestinal inflammation in this case (14, 16).

In conclusion, we described a case of intestinal BD that was successfully treated with IFX. Our extensive qPCR analyses of colonic biopsy samples before and after treatment with PSL or IFX revealed alterations in the expression levels of cytokines and chemokines. Increased mRNA expression of IL-6 and FOXP3 was associated with the remission induced by IFX in this case with intestinal BD. Thus, treatment with IFX has dual roles in the suppression of chronic inflammation in BD. First, IFX treatment might promote regeneration of the epithelium through the induction of IL-6 expression (12). Second, IFX treatment might suppress inflammation through the activation of FOXP3^+^ Tregs (7, 8). Alterations in IL-6 and FOXP3 mRNA expression levels seen in this case partially explain the molecular mechanisms by which IFX suppresses autoimmune reactions in intestinal BD. Future, studies utilizing a large number of patients with intestinal BD are required to verify this hypothesis.

## Data Availability Statement

The datasets presented in this article are not readily available because this is a case report and a dataset has not been generated. Requests to access the datasets should be directed to tomohiro@med.kindai.ac.jp.

## Ethics Statement

The studies involving human participants were reviewed and approved by Kindai University Faculty of Medicine. The patients/participants provided their written informed consent to participate in this study. Written informed consent was obtained from the patient for the publication of this report.

## Author Contributions

KY, IS, RT, AH, MKur, YM, YO, SM, and YK took care of the patient. KY and TW drafted the manuscript and performed the experiments. TY, KK, KM, and MKud edited and revised the manuscript. TC performed pathological analyses. All authors contributed to the article and approved the submitted version.

## Conflict of Interest

The authors declare that the research was conducted in the absence of any commercial or financial relationships that could be construed as a potential conflict of interest.
